# Resistance is futile: RNA-sequencing reveals differing responses to bat fungal pathogen in Nearctic *Myotis lucifugus* and Palearctic *Myotis myotis*

**DOI:** 10.1007/s00442-019-04499-6

**Published:** 2019-09-10

**Authors:** Thomas M. Lilley, Jenni M. Prokkola, Anna S. Blomberg, Steve Paterson, Joseph S. Johnson, Gregory G. Turner, Tomáš Bartonička, Erik Bachorec, DeeAnn M. Reeder, Kenneth A. Field

**Affiliations:** 1 Finnish Museum of Natural History, University of Helsinki, Helsinki, Finland; 2 Institute of Integrative Biology, University of Liverpool, Liverpool, UK; 3 Department of Biology, University of Turku, Turku, Finland; 4 Department of Biological Sciences, Ohio University, Athens, OH USA; 5 Pennsylvania Game Commission, Harrisburg, PA USA; 6 Department of Botany and Zoology, Masaryk University, Brno, Czech Republic; 7 Biology Department, Bucknell University, Lewisburg, PA USA

**Keywords:** Host–pathogen interaction, Resistance, Tolerance, Infection, Opportunistic pathogen

## Abstract

**Abstract:**

Resistance and tolerance allow organisms to cope with potentially life-threatening pathogens. Recently introduced pathogens initially induce resistance responses, but natural selection favors the development of tolerance, allowing for a commensal relationship to evolve. Mycosis by *Pseudogymnoascus destructans*, causing white-nose syndrome (WNS) in Nearctic hibernating bats, has resulted in population declines since 2006. The pathogen, which spread from Europe, has infected species of Palearctic *Myotis* for a longer period. We compared ecologically relevant responses to the fungal infection in the susceptible Nearctic *M. lucifugus* and less susceptible Palearctic *M. myotis*, to uncover factors contributing to survival differences in the two species. Samples were collected from euthermic bats during arousal from hibernation, a naturally occurring phenomenon, during which transcriptional responses are activated. We compared the whole-transcriptome responses in wild bats infected with *P. destructans* hibernating in their natural habitat. Our results show dramatically different local transcriptional responses to the pathogen between uninfected and infected samples from the two species. Whereas we found 1526 significantly upregulated or downregulated transcripts in infected *M. lucifugus*, only one transcript was downregulated in *M. myotis*. The upregulated response pathways in *M. lucifugus* include immune cell activation and migration, and inflammatory pathways, indicative of an unsuccessful attempt to resist the infection. In contrast, *M. myotis* appears to tolerate *P. destructans* infection by not activating a transcriptional response. These host-microbe interactions determine pathology, contributing to WNS susceptibility, or commensalism, promoting tolerance to fungal colonization during hibernation that favors survival.

**Graphic abstract:**

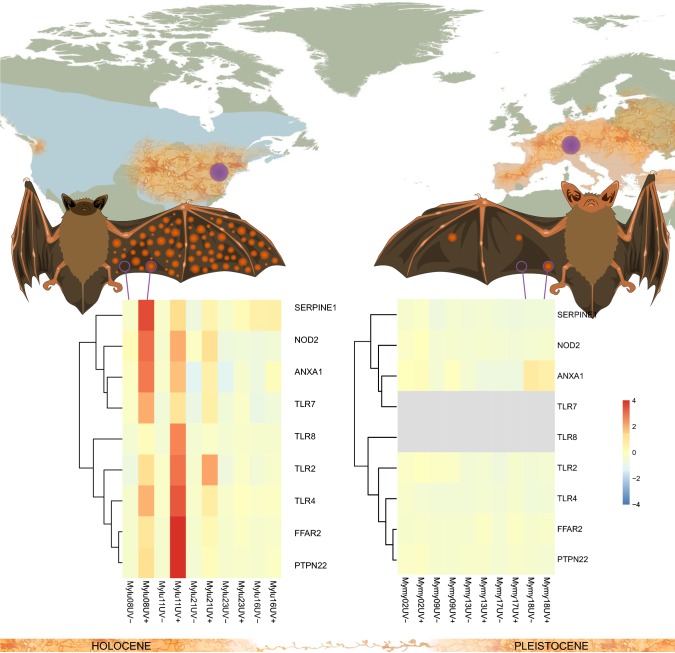

**Electronic supplementary material:**

The online version of this article (10.1007/s00442-019-04499-6) contains supplementary material, which is available to authorized users.

## Introduction

Exposure to novel pathogens in naive wildlife populations has increased over the last decades (Daszak et al. 2000). Many of these introductions are caused by humans inadvertently transporting microbes with pathogenic potential to new geographic locations, where they emerge as a major concern for native species (Tompkins et al. 2015). That is, the disease often occurs when there is a host shift or a change in host ecology, or in environmental conditions (Scholthof 2007). With chytridiomycosis, the worldwide fungal amphibian epidemic, disease was related to the emergence of a hypervirulent strain of fungus (Eskew and Todd 2013). In contrast, white-nose syndrome (WNS), a bat fungal disease, has been linked to varied host responses rather than increased pathogen virulence (Field et al. 2015; Leopardi et al. 2015; Bandouchova et al. 2018). WNS is caused by *Pseudogymnoascus destructans*, and has resulted in extensive declines in populations of several bat species since arriving in North America likely from Eurasia in 2006 (Blehert et al. 2009; Frick et al. 2010; Leopardi et al. 2015; Campana et al. 2017).

The psychrophilic fungus, *P. destructans* infects insectivorous bat hosts during hibernation. In Nearctic bats, the associated pathology leads to increased arousal frequency, consuming valuable energy reserves (Warnecke et al. 2012; Reeder et al. 2012). The detrimental effects of infection by *P. destructans* vary geographically and between hosts (Zukal et al. 2014, 2016; Bernard et al. 2015; Lilley et al. 2018; Bandouchova et al. 2018); bats in the genus *Myotis*, such as *M. lucifugus* and *M. septentrionalis*, appear to be especially susceptible. In *M. lucifugus*, mechanisms leading to pathology are expressed through a cascade of physiological responses (Verant et al. 2014; Field et al. 2015, 2018) and the immune response to the fungal infection is not able to provide protection (Johnson et al. 2015; Lilley et al. 2017). *Pseudogymnoascus destructans* is endemic in European bat hibernacula (Wibbelt et al. 2010; Martinkova et al. 2010; Puechmaille et al. 2011), where the fungus and cupping erosions in wing tissue of hosts, a diagnostic feature of infection by *P. destructans,* are found in at least 13 countries (Meteyer et al. 2009), in at least 15 species of bats (Zukal et al. 2016). Similar to North America, in Europe it appears that species in the genus *Myotis* are the most likely hosts, exhibiting similar tissue damage to Nearctic species (Zukal et al. 2016). However, despite the presence of *P. destructans* in the environment, and even invasion of host tissue, i.e. pathology, there are no signs of mass mortality in contemporary Palearctic bat populations (Wibbelt et al. 2010; Martinkova et al. 2010; Puechmaille et al. 2011; Pikula et al. 2012; Bandouchova et al. 2015). In fact, populations of bats preferentially hibernating at underground sites appear to be increasing (Van der Meij et al. 2015). However, mass accumulations of skeletal remains of *Myotis* bats in European cave deposits dating to the Pliocene and Pleistocene (c. 1.8-3.6 MYA) suggest a mass mortality event in the past (Martinkova et al. 2010). These mass accumulations may have been related to die-offs associated with WNS suggesting the Palearctic clade of *Myotis* would have gone through a selective event and have since coexisted with the pathogen (Harazim et al. 2018). The Palearctic and Nearctic Myotis clades diverged millions of years prior to this hypothesized event, approximately 12.2 MYA, consistent with North American bats remaining unexposed to the pathogen until recently (Ruedi et al. 2013).

Palearctic strains of *P. destructans* are able to infect Nearctic bats (Warnecke et al. 2012). Although strains on both continents show significant genetic similarity (Leopardi et al. 2015), Palearctic bats exposed to the North American strain appear to not get infected under captive conditions (Davy et al. 2017; Field et al. 2018a, b). It is unlikely that the high mortality caused by WNS in Nearctic bats is caused by a hypervirulent strain, such as in chytrid mycosis (Eskew and Todd 2013). Rather, it is differences in host susceptibility that appear to explain the different outcomes between Palearctic and Nearctic species (Bandouchova et al. 2018), which is supported by the overwintering success of Palearctic bats under similar pathogen pressure as their infected Nearctic counterparts (Zukal et al. 2016).

Palearctic bats, such as *Myotis myotis*, have been shown to tolerate infection by *P. destructans* (Bandouchova et al. 2018; Zukal et al. 2016). In contrast to resistance, which protects the host by actively reducing the pathogen burden, tolerance limits the harm caused by the pathogen, but has a neutral or even positive effect on the prevalence of the pathogen in the host population, as witnessed in Palearctic bat populations expressing high fungal loads, almost 100% prevalence, and only moderate pathology (Råberg et al. 2009; Martinkova et al. 2010; Zukal et al. 2014, 2016). Palearctic species of *Myotis* may have coevolved with *P. destructans* and the fungus may now exhibit a commensal or parasitic relationship with these less susceptible species.

Hibernating *M. lucifugus* severely infected with *P. destructans* show large, local transcriptional responses in genes associated with immune function during the intermittent euthermic bouts occurring throughout hibernation, whereas the local transcriptional response to infection is very low during torpor Field et al. (2018). The inflammatory responses, occurring only during arousals Field et al. (2018), maybe maladaptive, and the immunopathology related to infection could be a major driver of mortality associated with WNS, at least in *M. lucifugus* (Lilley et al. 2017). Recent reports have described populations of *M. lucifugus* in northeastern North America that are beginning to stabilize at substantially reduced population sizes, or even showing signs of population increase since the initial mass mortality following the arrival of WNS (Langwig et al. 2017; Dobony and Johnson 2018). This has led to speculation that this could be due to strong selection for those individual bats that responded to *P. destructans* with tolerance rather than a detrimental overresponse to fungal infection (Frick et al. 2017). Indeed, the balance between commensalism and pathogenesis is critical in many fungal diseases (Iliev and Underhill 2013). Interestingly, these remnant populations show high pathogen loads and signs of host tissue invasion, but no associated increase in arousal frequency, which is suggestive of tolerance (Lilley et al. 2016). There is, therefore, an important need to study the mechanisms of host survival in both Palearctic and survivor populations of Nearctic bats, as comparing host responses in these regions may provide clues to how Nearctic bats might be able to adapt to the pathogen, and what responses may result in continued mortality in Nearctic populations.

Here, we take a whole-transcriptome approach to compare responses of wild individuals of two species of *Myotis* commonly infected with *P. destructans* during the hibernation period: the Palearctic *M. myotis*, with a lengthy coexistence with the pathogen, and the Nearctic *M. lucifugus* from a remnant survivor population in Pennsylvania, United States, exposed to *P. destructans* c. 2009. Although a common garden approach should ideally be favored, we adopted to use wild animals in their natural habitat, because infection of captive *M. myotis* is difficult (Field et al. 2018a, b) and may lead to unrelated infections (Moore et al. 2018; Abbott et al. 2019). A previous study that attempted to address this question (Davy et al. 2017) was unable to measure any response to infection in captive *M. myotis* because the samples did not contain detectable levels of *P. destructans* (Davy et al. 2017). Therefore, we used non-lethal samples collected from bats hibernating in their natural habitat, and thus showing ecologically relevant responses to the fungal infection during their intermittent arousals, to determine how transcriptional responses to confirmed *P. destructans* infection differed in the two studied species. A sample from a location with fungal growth and another from a location with no growth was collected from euthermic bats during arousal from hibernation (Field et al. 2018). We predict that euthermic transcriptional responses in wing tissue during the hibernation period will reflect the differences in past exposure to the pathogen between the two species. In addition, we compare the results to a similar, previous study conducted on naïve *M. lucifugus* in their first year of coming into contact with the pathogen (Field et al. 2018). Tolerance to *P. destructans* is predicted to produce transcriptomic responses that either differs little between infected (UV-positive) and control (UV-negative) samples or show upregulationof anti-inflammatory and tissue repair pathways in infected tissue compared to control tissue (Soares et al. 2017; Medzhitov et al. 2012).

## Methods

### Ethical statement

Animals in the U.S. were collected and studied with Pennsylvania Game Commission Special Use Permit 33085. Sample collection protocols were approved by Bucknell University Institutional Animal Care and Use Committee (IACUC# DMR-16) in accordance with guidelines set forth by the USDA and PHS Policy on Humane Care and Use of Laboratory Animals under the guidance of the Office of Laboratory Animal Welfare (OLAW). The institution has an Animal Welfare Assurance on file with the NIH Office for the Protection of Research Risks (OPRR), Number A3525-01. Fieldwork and bat sampling in the Czech Republic were performed in accordance with Czech Law No. 114/1992 on Nature and Landscape Protection, based on permits 1662/MK/2012S/00775/MK/2012, 866/JS/2012 and 00356/KK/2008/AOPK issued by the Agency for Nature Conservation and Landscape Protection of the Czech Republic. Experimental procedures were approved by the Ethical Committee of the Czech Academy of Sciences (No. 16256/2015-MZE-17214). The author of the present study (TB) was authorized to handle free-living bats in agreement with Czech Certificate of Competency No. CZ01297 (§17, Act No. 246/1992).

### Sample collection

To compare the response of hosts to infection, we collected wing tissue samples from bats infected with *P. destructans*. For each bat, a pair of samples were collected: one sample was from a region of the wing with evidence of fungal growth and a second sample was from a region of the wing without evidence of fungal growth. These paired samples were obtained from adult wild male *M. lucifugus* (*N* = 5) and *M. myotis* (*N* = 5) during the last quartile of the hibernation period (18 Mar 2018 and 20 Mar 2018, respectively). The *M. lucifugus* samples were collected from a hibernaculum in Woodward, Pennsylvania, where population numbers have begun to increase in the past few years after initially declining by 90% due to WNS (GR Turner, pers. comment). The *M. myotis* samples were collected from the Simon a Juda mine, Czech Republic, with temporally stable population sizes. At both sites, torpid bats were collected from the walls, and their wings were immediately UV-transilluminated and photographed (Turner et al. 2014). A single fluorescing area (UV-positive), indicating infection of host tissue by *P. destructans*, was circled on the right wing using a sharpie and a non-fluorescent (UV-negative), control area was circled on the left wing (Fig. S1). Although *P. destructans* causes a local immune response (vs. systemic, Field et al. 2015) at infected sites, we prefer to use UV-positive and UV-negative to describe to distinguish the sample types, seeing as ultimately pathology occurs at the individual level. The bats were allowed to arouse from torpor for 60–120 min to initiate responses (Lilley et al. 2017; Field et al. 2018). After arousal, the circled areas were sampled using 5 mm biopsy punches (MLT3335, Miltex Instrument Co, Plainsboro, New Jersey) and placed in RNAlater (ThermoFischer, Waltham, Massachusetts). After this, the bats were sexed, the forearms were measured and their mass was recorded. The bats were released after the procedures. The samples were left at ambient temperature for 6–8 h to allow permeation of RNAlater into the tissue, after which the samples were transferred to a − 80 °C freezer for storage.

### Quantifying WNS-lesions

To quantify the severity of fungal infection, we transilluminated the wing membranes of each bat using a UV lamp emitting light at a wavelength of 368 nm (Turner et al. 2014). Each wing was photographed while transilluminated, and the number of fluorescent spots on each wing was calculated from the photographs (Figure S1). According to (Pikula et al. 2017), the number of UV-fluorescent lesions correlates with WNS pathology, demonstrating congruence between WNS-associated tissue damage and the extent of UV fluorescence. The number of lesions, calculated from photographs at the time of sampling, were considerably lower in *M. myotis* compared to *M. lucifugus* (Table S1).

### RNA extraction and sequencing

To avoid batch effects arising from RNA extractions, samples from the two species were extracted in mixed batches, with both wing punches from the same individual extracted in the same batch. RNA was extracted with Qiagen RNeasy Micro kit (Qiagen, Hilden, Germany), including a DNase I treatment. Samples were homogenized using motorized plastic pestles in 300 µL buffer RLT with ß-mercaptoethanol, after which the manufacturer’s protocol was followed. RNA was eluted in nuclease-free water and stored at − 80 °C. Samples were checked for quality using the Pico chip in Bioanalyzer 2100 (Agilent Technologies, Santa Clara, California). RNA Integrity values ranged from 5.7 to 8.9 and were on average 6.7 and 8.3 for *M. lucifugus* and *M. myotis*, respectively. RNA sequencing was performed by the University of Liverpool Centre for Genomic Research. Poly A-tailed RNA was enriched from total RNA samples using two rounds of selection with NEBNext Poly(A) mRNA Magnetic Isolation Module and assessed by Bioanalyser. RNA–Seq libraries were prepared from the Poly A selected material using the NEBNext Ultra Directional RNA Library Prep Kit for Illumina. Each library was quantified using Qubit and the size distribution assessed using the Bioanalyzer. These final libraries were pooled in equimolar amounts using the Qubit and Bioanalyzer data. The quantity and quality of each pool were assessed by Bioanalyzer and subsequently by qPCR using the Illumina Library Quantification Kit from Kapa (KK4854) on a Roche Light Cycler LC480II according to manufacturer’s instructions. The libraries were sequenced across two lanes of the HiSeq 4000 at 2 × 100 bp paired-end sequencing and produced an average of 29 million reads per sample. The raw FASTQ files were trimmed for the presence of Illumina adapter sequences using Cutadapt version 1.2.1. (Martin 2011). The reads were further trimmed using Sickle version 1.200 (Joshi and Fass 2011) with a minimum window quality score of 20.

### Gene expression

Prior to analysis of host gene expression, transcript levels of *P. destructans* were determined by alignment of trimmed reads to the concatenated genomes of *M. lucifugus* (Myoluc2.0, Ensembl release 84 (Yates et al. 2015) and *P. destructans* (Drees et al. 2016) with STAR v.2.6.1a (Dobin et al. 2013) and counts estimated with RSEM v1.3.1 (Li and Dewey 2011). Quantification of *P. destructans* transcript expression in transcripts per million (TPM) was used to determine the level of infection in each sample. The *P. destructans* transcripts were then removed from further analysis. Mapping rates to *M. lucifugus* were higher for the samples from *M. lucifugus* (85.7% ± 0.6%) than for *M. myotis* (76.9% ± 0.8%) (Table S2). Sample quality control and differential gene expression were then assessed using SARTools v.1.6.6 (Varet et al. 2016) and edgeR v.3.22.3 (Robinson et al. 2010). We used the scottyEstimate function of Scotty (Busby et al. 2013) to measure the statistical power of the differential expression study design with the following parameters: fc = 2, pCut = 0.05, minPercDetected = 50, costPerRepControl = 140, costPerRepTest = 140, costPerMillionReads = 10, totalBudget = 10,000, maxReps = 10, minReadsPerRep = 10,000,000, maxReadsPerRep = 100,000,000, minPercUnbiasedGenes = 50, pwrBiasCutoff = 50, and alignmentRate = 75. Scotty analysis was performed in Matlab R2018a (9.4.0.813654).

Prior to differential expression testing, transcripts were filtered with a cutoff after TMM-normalization of 1 TPM in at least 5 samples. A generalized linear model was used to fit the TMM-normalized transcript counts using the individual as a batch effect (~ individual + infection). Interactive MA plots were generated using Glimma v.1.10.1 (Su et al. 2017). Similar results were obtained using DESeq 2 v.1.20.0 (Love et al. 2014, p. 2) (Figure S2).

For *M. lucifugus*, gene ontology annotations were from Ensembl release 94 (Yates et al. 2015) and gene ontology enrichment analysis was performed using g:Profiler (Reimand et al. 2016) g:GOSt v.e94_eg41_p11_50c103b with a g:SCS threshold of 0.05. Enrichment was measured using ranked lists (by FDR) against the background of all annotated *M. lucifugus* genes. REVIGO (Supek et al. 2011) was used to filter the gene ontology categories for redundancy.

For calculating alignment rates to other genomes, STAR v.2.6.1 in quant mode was used to align reads to either *M. davidii* genome RefSeq assembly GCF_000327345.1 or *M. brandtii* RefSeq assembly GCF_000412655.1 (NCBI). For comparisons between the dataset generated in this study and our previous study of captive *M. lucifugus* (*n* = 6) from Wisconsin Field et al. (2018), we used the comBat function in the sva package v.3.28.0 (Johnson et al. 2007). This dataset is available as PRJNA393517 at the NCBI Sequence Read Archive. Paired samples of UV-negative and UV-positive biopsies were collected from *M. lucifugus* infected with *P. destructans* in captivity and sampled 70-80 min after emergence from torpor.

## Results

This study set out to test the hypothesis that *M. myotis* exhibits similar whole-transcriptome responses to *P. destructans* infection as the *M. lucifugus* from remnant populations that are more recently exposed to the pathogen. We predicted that differences between the host transcriptomic responses between these two species would illuminate the mechanism for the reduced susceptibility of *M. myotis* to WNS. To verify that the *M. myotis* samples that we had obtained were infected with *P. destructans*, we first tested the strict a priori assumption that *P. destructans* transcript levels would be present at relative levels at least as high in *M. myotis* samples as in *M. lucifugus* samples.

**Fig. 1:**
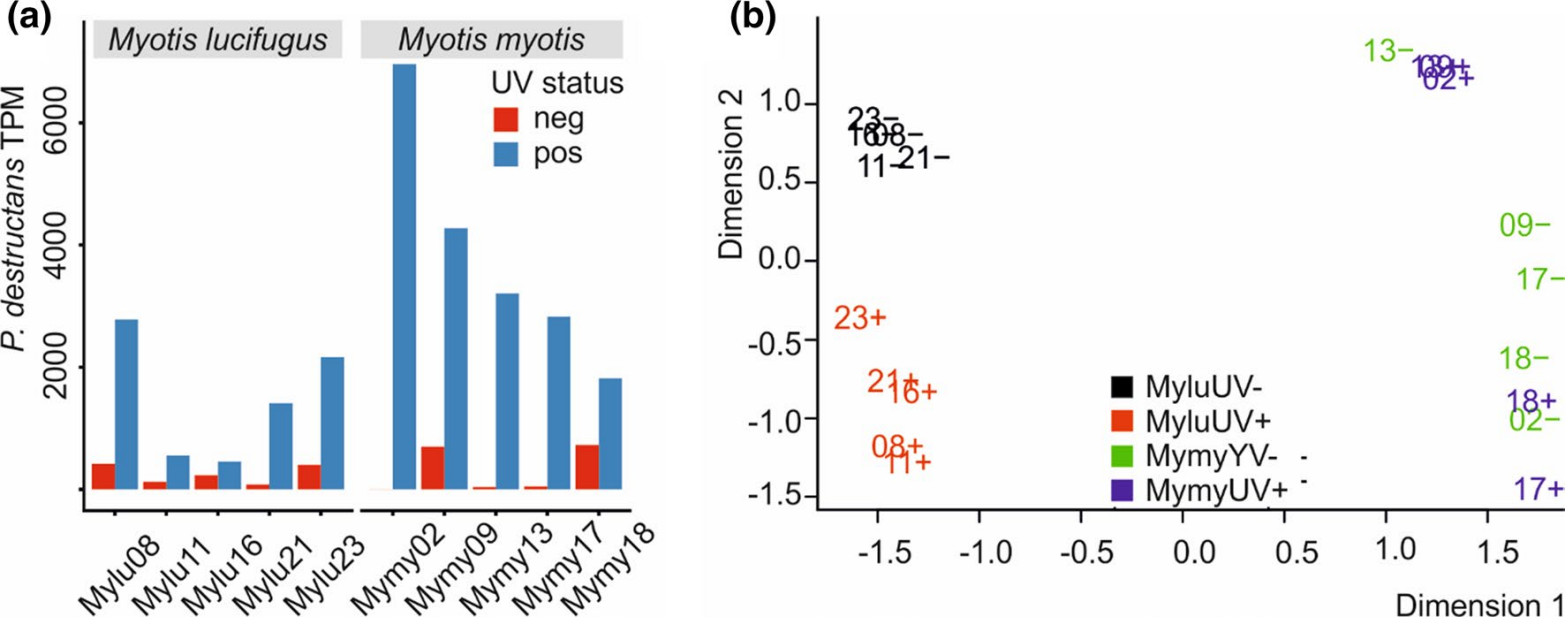
**a** Loads of *P. destructans* in each sample determined by read mapping, **b** Principal component analysis of host transcript expression after removal of *P. destructans* reads. The load of *P. destructans* in each biopsy was determined by estimating *P. destructans* transcript counts in transcripts per million mapped reads (TPM). The ﻿multidimensional scaling plot shows pairwise comparisons of *M. lucifugus* transcript expression using moderated log CPM expression levels. Samples are colored by group, as indicated on the legend. Dimension 1 represents 49% of the variance dimension 2 represents 25% of the variance

To compare the local whole-transcriptomic responses of *M. lucifugus* and *M. myotis* to *P. destructans* infection, we obtained paired wing-tissue biopsies from bats of both species. Although the total number of lesions was lower in *M. myotis* (Table S1), we verified that an approximately equal amount of *P. destructans* was present in each group of biopsy samples by mapping of RNA-Seq reads (Fig. 1a). We found that UV-positive *M. lucifugus* contained 1475 ± 1010 *P. destructans* transcripts per million mapped reads (TPM) and UV-positive *M. myotis* 3817 ± 1965 TPM (Welch two-sample *t* test, p = 0.0557). The UV-negative samples from both species contained significantly less *P. destructans* than the UV-positive samples (278 ± 273, paired t-test p = 0.028 for *M. lucifugus*, p = 0.0044 for *M. myotis*). The numbers of reads that mapped to *P. destructans* in the UV-negative samples was comparable to tissue samples from bats that have never been exposed to *P. destructans* Field et al. (2018a, b) and may represent other fungi with homologous transcripts. The relative levels of *P. destructans* reads in the UV-positive samples were lower than we have found in a previous study of *M. lucifugus* infected in captivity Field et al. (2018) and this precluded analysis of differential gene expression in *P. destructans* genes.

After removing reads that mapped to *P. destructans*, we compared host gene expression patterns for *M. lucifugus* and *M. myotis*. Principal component analysis showed that the two species were distinct in their gene expression patterns (Fig. 1b). We also found that the UV positive and UV negative *M. lucifugus* samples showed distinct patterns of gene expression, as expected, but this clustering was not observed for the *M. myotis* samples.

**Fig. 2:**
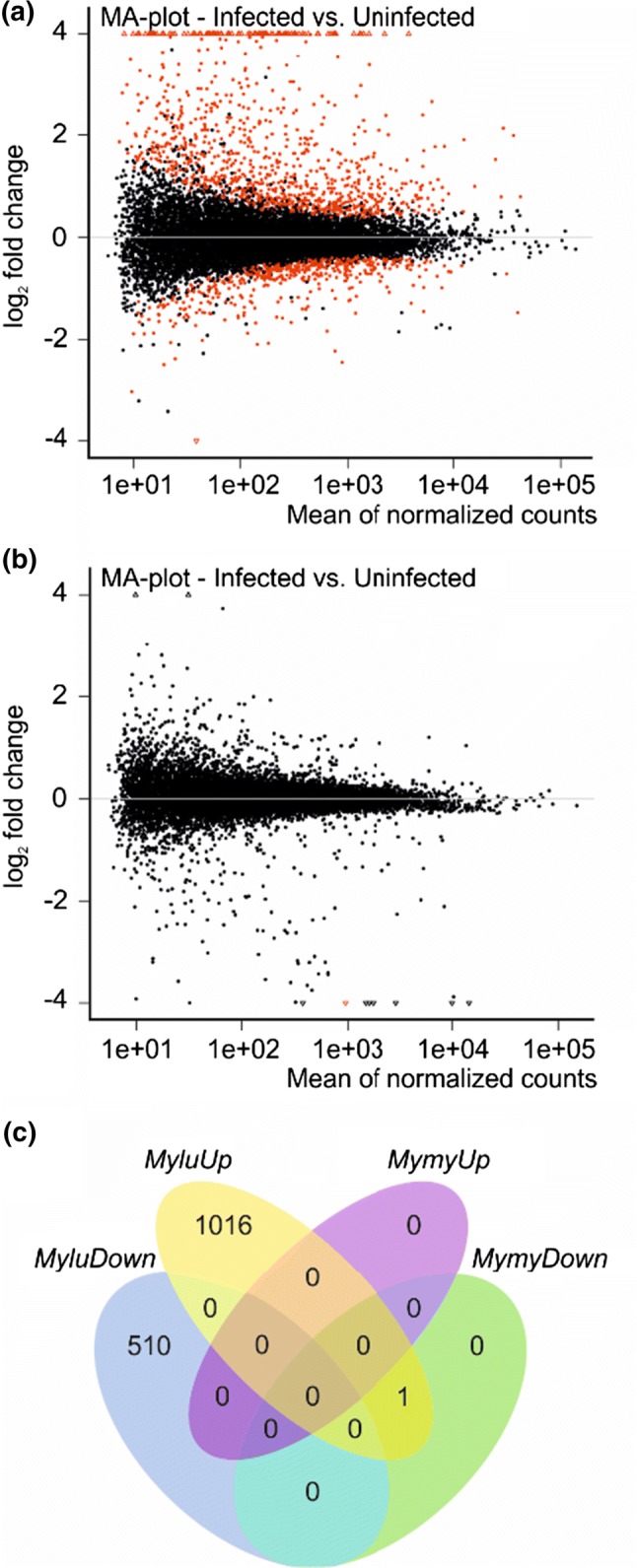
Differential expression of transcripts in tissues infected with *P. destructans* in **a***M. lucifugus*, **b** in *M. myotis* and **c** a comparison of expression in both host species. The mean expression level (log_2_ counts per million (CPM)) and the fold change (log_2_ FC) are shown for each transcript. Red points indicate differential expression (FDR ≤ 0.05 determined by edgeR). An interactive version of **a** is available at https://digitalcommons.bucknell.edu/fac_pubs/133/ and **b** at https://digitalcommons.bucknell.edu/fac_pubs/134/. The Venn diagram indicates the overlapping subsets of significantly differentially expressed transcripts (FDR < 0.05) in *M. lucifugus* (Mylu) and *M. myotis* (Mymy) samples

For *M. lucifugus*, the local changes in gene expression due to infection with *P. destructans* (Fig. 2a, Table 1) were similar in magnitude to what we observed in prior studies of this species Field et al. (2018). However, *M. myotis* showed much lower fold changes in gene expression due to local infection with *P. destructans*, despite similar levels of infection within the biopsied samples (Fig. 2b). Only one transcript was differentially expressed in *M. myotis* using the a priori FDR cutoff of 0.05, while 1526 transcripts were differentially expressed in the *M. lucifugus* samples (Fig. 2c, Supplemental dataset 1). Similar results were obtained if DESeq 2 was used instead of edgeR or if transcripts were mapped to *M. davidii* (72.2% + − 0.8% for *M. lucifugus* samples and 75.0% + − 0.6% for *M. myotis* samples) or *M. brandtii* (80.9% + − 0.8% for *M. lucifugus* samples and 75.5% + − 0.5% for *M. myotis* samples) transcriptomes instead of *M. lucifugus* (Figures S3B and S3C). The lack of differential expression in *M. myotis* was not due to low levels of mapping to the *M. lucifugus* transcriptome. Of the 1526 transcripts differentially expressed in *M. lucifugus*, 1396 (91.5%) were expressed sufficiently in *M. myotis* to pass the expression cutoff. Using Scotty (Busby et al. 2013), we determined that the study design, with 5 replicates per group, was sufficiently statistically powerful to detect at least 50% of expressed genes that are differentially expressed by a 2X fold change at *p* < 0.05 (Figure S4). Together, these results indicate that our study was sufficiently powerful to detect differential gene expression in *M. myotis* if it had been present.

**Table 1:** Select immune genes differentially expressed in UV-positive tissue with *P. destructans* in *M. myotis* and *M. lucifugus*

Transcript	Name	Description	*M. lucifugus*		*M. myotis*	
			FC	FDR	FC	FDR
ENSMLUT00000015856	IL6	Interleukin 6	9.58	9.65e-26	NA	NA
ENSMLUT00000011363	CCL2	C–C motif chemokine 2	7.46	5.92e-25	1.67	1
ENSMLUT00000008206	PTGS2	Prostaglandin-endoperoxide synthase 2	15.89	1.87e-20	1.27	1
ENSMLUT00000008807	HMOX1	Heme oxygenase 1	4.20	9.96e-17	1.21	1
ENSMLUT00000012289	ICAM1	Intercellular adhesion molecule 1	4.29	3.11e-16	1.57	1
ENSMLUT00000016420	THBS1	Thrombospondin 1	6.28	1.72e-13	1.06	1
ENSMLUT00000001355	MMP25	Matrix metallopeptidase 25	35.75	2.39e-13	2.19	1
ENSMLUT00000004880	CXCR2	C-X-C motif chemokine receptor 2	24.25	2.53e-13	2.60	1
ENSMLUT00000015164	NOD2	Nucleotide binding oligomerization domain containing 2	3.86	3.41e-13	1.20	1
ENSMLUT00000029244	TNFAIP6	TNF alpha induced protein 6	3.81	6.61e-13	1.18	1
ENSMLUT00000011581	CLEC4E	C-type lectin domain family 4 member E	29.04	7.63e-12	NA	NA
ENSMLUT00000000289	MMP9	Matrix metallopeptidase 9	12.21	1.38e-10	3.18	0.09
ENSMLUT00000002542	CXCL16	C-X-C motif chemokine ligand 16	2.35	1.49e-09	1.25	1
ENSMLUT00000000473	ANXA1	Annexin A1	2.87	1.55e-09	0.96	1
ENSMLUT00000008598	SELE	Selectin E	4.86	2.21e-09	0.93	1
ENSMLUT00000014922	SHB	SH2 domain containing adaptor protein B	2.06	7.73e-09	1.13	1
ENSMLUT00000012386	FFAR2	Free fatty acid receptor 2	14.72	9.26e-09	4.76	0.22
ENSMLUT00000003161	ITGB2	Integrin subunit beta 2	17.88	9.69e-09	2.71	0.54
ENSMLUT00000011719	NR4A3	Nuclear receptor subfamily 4 group A member 3	2.77	1.59e-08	1.00	1
ENSMLUT00000006594	RELB	RELB proto-oncogene, NF-kB subunit	2.14	1.73e-08	1.18	1
ENSMLUT00000002906	S100A8	S100 calcium binding protein A8	3.76	4.98e-08	1.29	1
ENSMLUT00000003286	SBNO2	Strawberry notch homolog 2	2.64	1.02e-07	1.15	1
ENSMLUT00000011146	TRPM2	Transient receptor potential cation channel subfamily M member 2	14.62	1.29e-07	NA	NA
ENSMLUT00000012815	TLR2	Toll like receptor 2	3.29	1.33e-07	1.13	1
ENSMLUT00000003912	TLR7	Toll like receptor 7	6.87	1.71e-07	NA	NA
ENSMLUT00000007434	PTPN22	Protein tyrosine phosphatase, non-receptor type 22	10.56	1.71e-07	1.41	1
ENSMLUT00000014401	LCP1	Lymphocyte cytosolic protein 1	19.03	2.19e-07	3.03	0.43
ENSMLUT00000008567	SELL	Selectin L	42.81	2.31e-07	3.34	1
ENSMLUT00000012752	TLR8	Toll like receptor 8	29.04	3.63e-07	NA	NA
ENSMLUT00000003440	CORO1A	Coronin 1A	6.77	4.03e-07	1.68	1
ENSMLUT00000008354	ITGAL	Integrin subunit alpha L	14.42	1.03e-06	NA	NA
ENSMLUT00000031197	S100A9	S100 calcium binding protein A9	4.38	2.01e-06	1.25	1
ENSMLUT00000000160	PTAFR	Platelet activating factor receptor	6.96	2.05e-06	2.01	1
ENSMLUT00000022221	JAML	Junction adhesion molecule like	4.06	2.22e-06	1.16	1
ENSMLUT00000001008	SERPINE1	Serpin family E member 1	3.53	2.72e-06	1.32	1
ENSMLUT00000015767	THY1	Thy-1 cell surface antigen	3.36	6.87e-06	1.03	1
ENSMLUT00000000245	TFRC	Transferrin receptor	2.46	1.01e-05	0.92	1
ENSMLUT00000014583	FGR	FGR protooncogene Src tyrosine kinase	8.57	1.66e-05	1.91	1
ENSMLUT00000007409	TLR4	Toll like receptor 4	3.84	2.07e-05	1.32	1
ENSMLUT00000008843	IL17C	Interleukin 17C	5.39	2.10e-05	NA	NA

Ensembl transcript ID and gene name are listed for selected transcripts differentially expressed in *M. lucifugus* between UV-negative and UV-positive tissue. The fold change (FC) and Benjamini–Hochberg adjusted p value (FDR) calculated by edgeR are shown for each transcript for samples from both *M. lucifugus* and *M. myotis* samples. Bold FDR values indicate ≤ 0.05. NA indicates transcripts that were removed by filtering for low expression level (TPM < 1 in 5 or more samples) prior to edgeR testing. See Table S1 for results for all transcripts

We next used gene ontology analysis to determine the functional categories that were enriched in the transcripts that showed differential expression due to local infection of the *M. lucifugus* samples. We found that many of the differentially expressed genes were involved in muscle cell development/function and immune responses (Supplemental dataset 2). Many of the most enriched categories involved the development and function of muscle cells (for example, GO:0055002, striated muscle cell development, adjusted *p* = 5.94 10^−15^), even after filtering for redundancy (Supplemental dataset S3). However, during exploratory data analysis we determined that the differential expression of these categories of muscle genes was due to the inadvertent bias of sampling biopsies from the plagiopatagium (wing tissue between the hindleg and the phalanges) or chiropatagium (wing tissue between the hindlegs and the tail). None of the *M. myotis* samples were obtained from the chiropatagium, while three *M. lucifugus* biopsies were from the chiropatagium, all in the UV negative group (Table S3). When we analyzed the *M. lucifugus* samples for differential expression based on biopsy location post hoc (Supplemental dataset 4), we found strong enrichment of muscle development and function gene ontology categories (for example, GO:0,055,002, striated muscle cell development, adjusted *p* = 1.07 × 10^−19^).

**Fig. 3:**
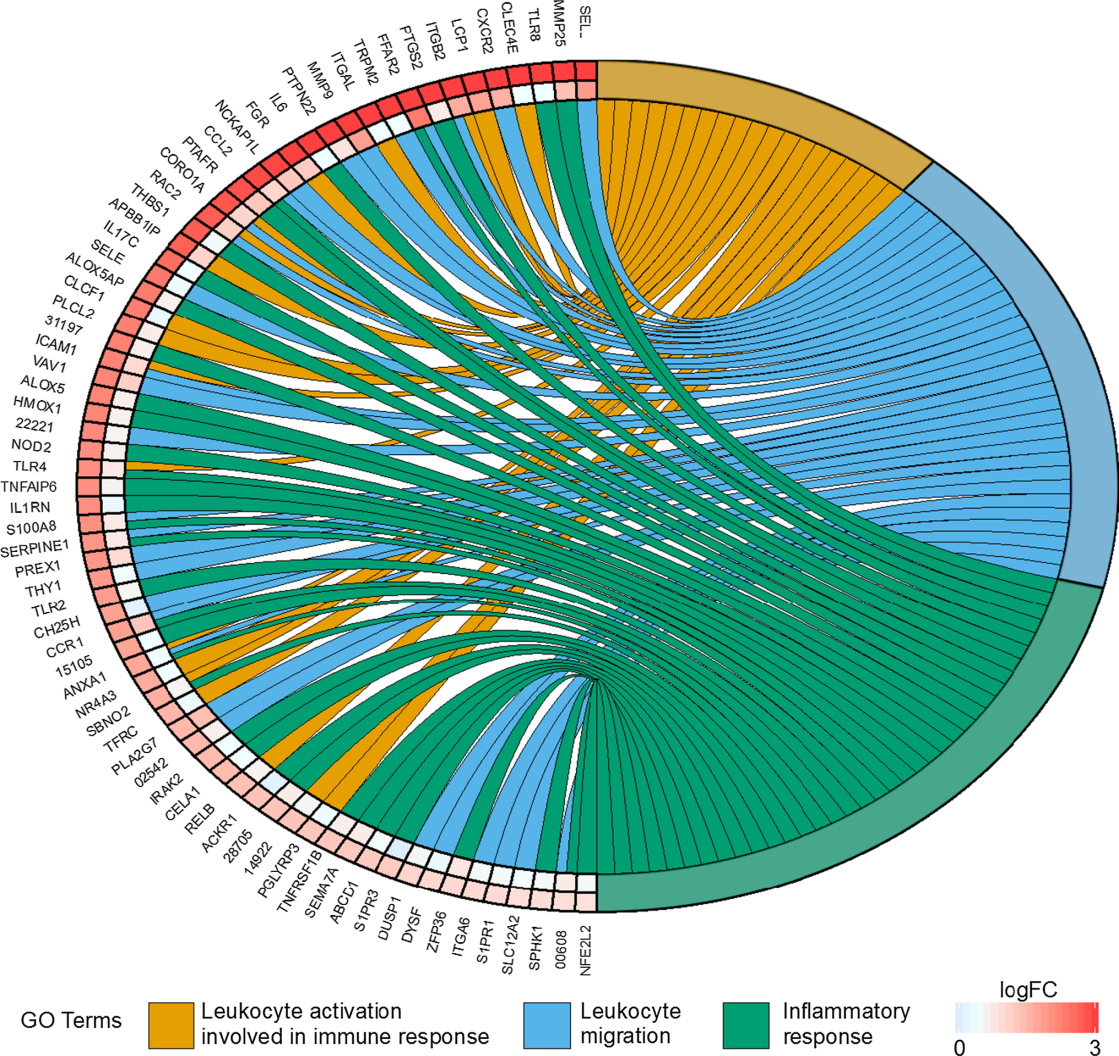
Chordplot of genes involved in immune responses in *M. lucifugus* identified by gene ontology analysis. Connections from the right side of the figure to the left signify associations between transcripts and selected biological process categories. All transcripts differentially expressed (FDR < 0.05 by edgeR) are shown that were annotated in the following categories: GO: 0,002,366 (leucocyte activation involved in immune response (orange)), GO: 0,050,900 (Leukocyte migration (blue)), and GO: 0,006,954 (Inflammatory response (green)). Expression level changes (log_2_ fold change) are shown for the comparison of UV-negative to UV-positive *M. lucifugus* (outer heatmap) and UV-negative to UV-positive *M. myotis* (inner heatmap) (color figure online)

The functional categories of genes enriched due to *P. destructans* infection in *M. lucifugus* included leukocyte activation involved in immune response (GO:0,002,366, adjusted p = 2.95 × 10^−5^), leukocyte migration (GO:0,050,900, adjusted *p* = 3.63 × 10^−5^), and inflammatory response (GO:0,006,954, adjusted *p* = 8.06 x10^−4^). All of the genes differentially expressed in these categories due to local infection in *M. lucifugus* showed lower fold-changes due to infection in *M. myotis* and were not differentially expressed in UV-positive tissue in *M. myotis* (Fig. 3 and Supplemental dataset 1) or correlated with biopsy location in *M. lucifugus* (Supplemental dataset 4).

**Fig. 4:**
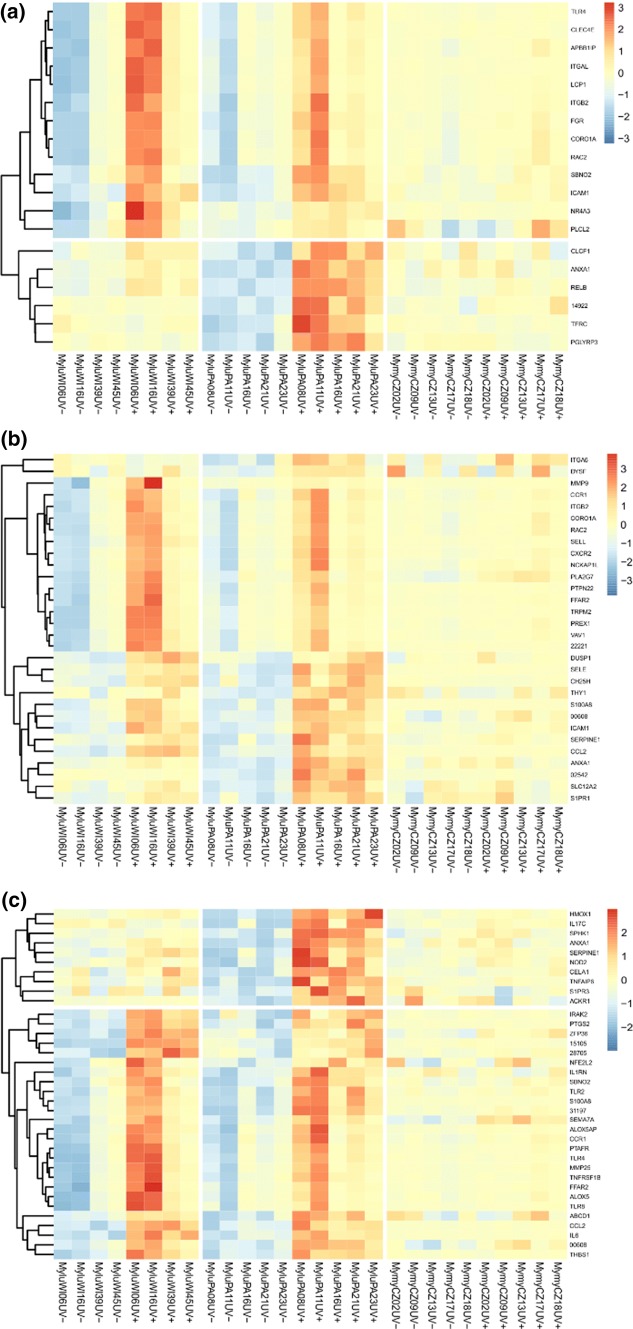
Normalized expression levels of transcripts among *M. lucifugus* (Wisconsin and Pennsylvania, left to right) and *M. myotis* involved in **a** leukocyte activation, **b** leukocyte migration and **c** inflammatory response. All transcripts differentially expressed in Pennsylvania *M. lucifugus* samples (FDR < 0.05 by edgeR) are shown that were annotated in the following categories: **a** GO:0,002,366 (leucocyte activation involved in immune response (orange)), **b** GO: 0,050,900 (Leukocyte migration (blue)), and **c** GO: 0,006,954 (Inflammatory response (green)) (color figure online)

In a previous study using the same paired sampling approach in captive bats Field et al. (2018), we found that *M. lucifugus* from a population of bats from Wisconsin, naïve to *P. destructans* exposure, showed robust local responses to *P. destructans* infection after arousal from torpor. To determine if the local responses to *P. destructans* differ in populations of bats with presumed prior exposure to *P. destructans* infection, we compared the differential expression of transcripts due to infection between the Wisconsin and Pennsylvania populations after correcting for individual variation (and any batch effects). We compared the expression of transcripts in the categories of genes identified from the gene ontology analysis of the current study and found differing patterns of differential expression between the naïve *M. lucifugus* from Wisconsin, the experienced *M. lucifugus* from Pennsylvania and the *M. myotis* from the Czech Republic (Fig. 4). We found that none of the genes in these categories were differentially expressed due to local infection in the *M. myotis* samples. In contrast, some of these genes showed very high levels of upregulation due to local infection in the *M. lucifugus* from Wisconsin and the pattern of expression levels varied in individual bats in ways that were distinct from the *M. lucifugus* from Pennsylvania.

## Discussion

Our study demonstrates a potential mechanism of tolerance as a host defense mechanism against pathogens (Boots and Bowers 1999; Miller et al. 2006; Kutzer and Armitage 2016; Soares et al. 2017; Ganeshan et al. 2019). Tolerance is a damage control mechanism that prevents the deleterious effects of pathogens and uncouples immune-driven resistance mechanisms from immunopathology and disease (Soares et al. 2017). In mice and humans, tolerance to malaria has been shown to depend on the anti-inflammatory properties of heme oxygenase-1 (Råberg et al. 2007; Ferreira et al. 2011). Immune responses to fungi are tuned to balance tolerance to environmental and commensal fungi and protective responses to pathogens (Iliev and Underhill 2013), and excessive tissue damage is prevented through modulation of interleukin-17 signalling (Song et al. 2015). Our results support an additional mechanism of tolerance that plays a role in the survival of WNS in *M. myotis*, dampening or delaying a damaging immune response until resources are available, after emergence from hibernation.

Although hosts may cope with infections through resistance responses and tolerance, only the latter is predicted to be favored by natural selection in the long-term and become eventually fixed (Roy and Kirchner 2000). Our data support this prediction. Populations of *M. lucifugus*, which have newly encountered *P. destructans*, suffer high mortality from infection and we show that *M. lucifugus*, even in so-called survivor populations individuals exhibit clear transcription profiles of immune gene expression. By contrast, *M. myotis*, which has coexisted with the pathogen for millennia, are commonly infected by the pathogen but exhibit no immune response to infection despite associated pathology. The study emphasizes the advantage of studying this wildlife disease in natural conditions instead of in laboratory settings, where the hosts are not prone to infection.

Our paired sampling protocol enabled a transcriptome-wide comparison of during-arousal, local responses of two bat species naturally infected with the fungal pathogen, *P. destructans*. The Palearctic *M. myotis* with a lengthy coexistence with *P. destructans* showed no significant transcriptional response to infection (one downregulated gene between comparisons). The relationship between the *M. myotis* host and the fungus may have evolved into a commensal relationship that allows the host to tolerate the infection without disrupting hibernation. The Nearctic remnant *M. lucifugus* population studied here, only subjected to the pathogen since c. 2009, showed abundant transcriptional responses (1526 significantly upregulated or downregulated transcripts) that include an upregulated immune response to the pathogen, presumably an attempt at resistance. In the light of catastrophic population declines in affected Nearctic species, this attempted resistance response could contribute to the increased arousals from torpor and emaciation associated with WNS pathology (Reeder et al. 2012; Meteyer et al. 2012; Warnecke et al. 2013; Lilley et al. 2017). Conversely, *M. myotis* appears to tolerate *P. destructans* infection during periodic arousals from torpor. Although other factors are also likely at play, the absence of a response in *M. myotis* suggests that this species could have adapted to endemic *P. destructans* exposure by tolerating infection, at least during periodic arousals, allowing for valuable energy reserves to be preserved during hibernation. This reduced investment in immune responses during hibernation is consistent with the energetic trade-offs that have been observed during daily heterothermy in mice (Ganeshan et al. 2019). Hibernation represents an extreme state of hypometabolism and the lack of local response to infection seen in *M. myotis* may represent a favorable energetic trade-off to conserve energy until emergence when a robust immune response is within the energy budget (Ganeshan et al. 2019).

We did not observe a response in *M. myotis* that involved the downregulation of inflammatory genes nor the upregulation of anti-inflammatory or tissue repair genes. Genes involved in modulation of the immune response, tissue regeneration, and wound healing show signals of positive selection in *M. myotis* (Harazim et al. 2018). It is possible that these classes of genes play an important role in WNS tolerance after emergence from hibernation (Meteyer et al. 2012; Meierhofer et al. 2018) and were not evident in our analysis within the first 60–120 min post-arousal.

Our sampling protocol was not designed to detect systemic changes in host gene expression Field et al. (2018), so we cannot rule out that *M. myotis* do respond to mycosis by altering gene expression in both UV-positive and UV-negative tissues or in other organs. However, given the complete absence of a response at the local level, we consider this unlikely. Although the transcriptional activity of the fungus was comparable between the species within the lesions themselves, *M. myotis* had fewer total lesions than *M. lucifugus*, suggesting the growth of *P. destructans* in *M. myotis* could be controlled by other factors not associated with transcriptional host responses during arousals. These factors may include abiotic environmental conditions (Johnson et al. 2014), microbial competition (Cornelison et al. 2014; Micalizzi et al. 2017), and the antifungal properties of epidermal fatty acid esters (Frank et al. 2018).

The two main groups of enriched genes in the UV-positive *M. lucifugus* samples were associated with muscle cells and immune responses. The muscle development and function genes correlate with sampling location and do not appear to be associated with *P. destructans* infection; the plagiopatagium has muscles, whereas the chiropatagium does not. The immune response pathways that are upregulated include immune cell activation, migration, and inflammatory pathways. These results are very similar to those seen in responses to *P. destructans* in *M. lucifugus* that had not encountered the fungus previously Field et al. (2015, 2018). Genes with putative immune function, such as *IL6*, *CCL2*, *PTGS2*, *ICAM1*, *MMP25*, *CLEC4E*, *FFAR2*, and *SELL* have also been found to be upregulated at both the local Field et al. (2018) and systemic levels (Field et al. 2015) during WNS. The local response in *M. lucifugus* appears to be highly inflammatory and includes pro-inflammatory cytokines, chemokines, and damage-associated molecular pattern recognition molecules. There are 11 putative S100 protein transcripts significantly upregulated in *M. lucifugus* (Supplemental dataset 1) and none of these transcripts were significantly upregulated in the *M. myotis* samples. These S100 proteins are part of an inflammatory response to the pathogen, but one that is pathological because of the magnitude and/or the timing of the response during the hibernation period.

Although the bats sampled from a remnant population in Pennsylvania, in which the population of bats have survived with *P. destructans* exposure for almost 10 years, share many differentially expressed genes with the naive population in Wisconsin, there are some interesting differences. Some genes involved in immune responses show greater local responses to *P. destructans* infection, while others show attenuated responses (Fig. 4). This may indicate that a different type of response has already been selected for in bats in the Pennsylvania populations (Johnson et al. 2016; Cheng et al. 2019), which also showed greater variation in responses between individuals. Alternatively, selection for phenotypic plasticity in their response may allow individual bats to persist over time in the face of WNS.

It appears that remnant populations of *M. lucifugus* in North America are responding to the mycosis caused by *P. destructans* differently to naïve populations, which are coming into contact with the pathogen for the first time. However, even though the response may have shifted in the remnant bats, it is still very different to the complete lack of response in the Palearctic *M. myotis*. Whether the shift in response to mycosis in the remnant populations contributes to survival needs to be further assessed quantitatively in conjunction with other factors that have been found to contribute to survival (Johnson et al. 2014; Frick et al. 2017; Micalizzi et al. 2017, p. 2; Frank et al. 2018; Cheng et al. 2019). However, with small host population sizes affected by an opportunistic environmental pathogen, the possibility of stochastic effects on these remnant populations should be of great concern as climate change continues to escalate (Gallana et al. 2013; European Environment Agency 2016) and the biomass of the diet these bats depend on, insects (Hallmann et al. 2017) has begun to dwindle.

The results from this study support a model that Nearctic bats when first encountering the novel *P. destructans* pathogen, exhibit a pathological response. There are several important implications of this model. First, the response to *P. destructans* infection should attenuate over time as the result of selective pressure or phenotypic plasticity. We may be seeing some initial evidence of this adaptation in North America [Fig. 4 (Lilley et al. 2016; Langwig et al. 2017; Cheng et al. 2019)]. Second, WNS intervention strategies designed to heighten the host response to *P. destructans* infection may lead to increased pathology. This may explain the difficulty in designing an effective vaccine or other treatment methods (Johnson et al. 2014; Lilley et al. 2017; Rocke et al. 2019), and suggests that caution should be taken in testing such an approach. Third, our results point to the need for further study of the gene expression responses in additional populations and species of Nearctic and Palearctic bats. If it is true that species, populations, and individuals that tolerate infection are able to better cope with WNS, then further studies can help predict the fate of bats in the face of this, and other, anthropogenic challenges.

## Electronic supplementary material

Below is the link to the electronic supplementary material.

Supplementary material 1 (XLSX 5190 kb)

Supplementary material 2 (XLSX 43 kb)

Supplementary material 3 (CSV 12 kb)

Supplementary material 4 (XLSX 20 kb)

Supplementary material 5 (DOCX 819 kb)

## Data Availability

Sequence data is available through NCBI via SRA accession PRJNA564421.
