# Supplementary material for: Resistance is futile: RNA-sequencing reveals differing responses to bat fungal pathogen in Nearctic Myotis lucifugus and Palearctic Myotis myotis
Source: Oecologia. 2019 Sep 10;191(2):295–309. doi: 10.1007/s00442-019-04499-6 (PMC6763535; doi:10.1007/s00442-019-04499-6)
Supplement: Supplementary file 5 — Supplementary material 5 (DOCX 819 kb) [file 442_2019_4499_MOESM5_ESM.docx]

Supplementary Information for:

“Resistance is futile: RNA-sequencing reveals differing responses to bat fungal pathogen in Nearctic *Myotis lucifugus* and Palearctic *Myotis myotis*

Including:

**Figs. S1 to S4**

**Tables S1 to S3**

**Supplemental datasets 1 -4 as separate files.**


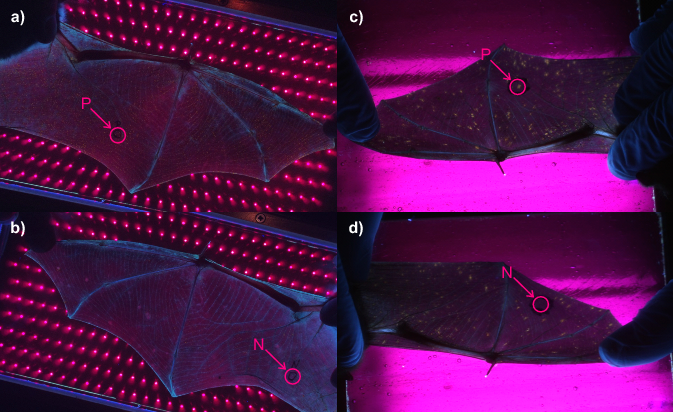


Fig. S1. Examples of sampling locations on bats: a) UV-positive *M. myotis* b) UV-negative *M. myotis* c) UV-positive *M. lucifugus* d) UV-negative *M. lucifugus*.


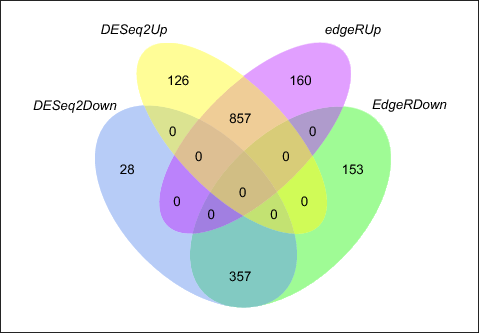


Fig. S2. Comparison of differential expression in *M. lucifugus* using DESeq2 and edgeR.


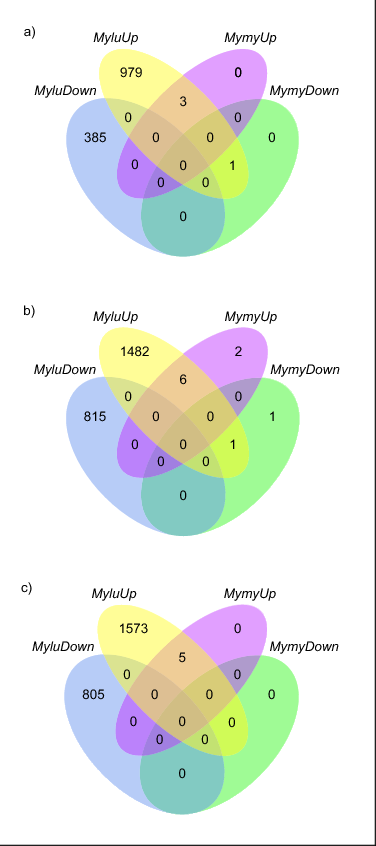


Fig. S3. Comparisons of differential expression using edgeR between *M. lucifugus* and *M. myotis* when transcripts are mapped to a) *M. lucifugus* genome, b) *M. davidii genome*, b) *M. brandtii* genome.

**
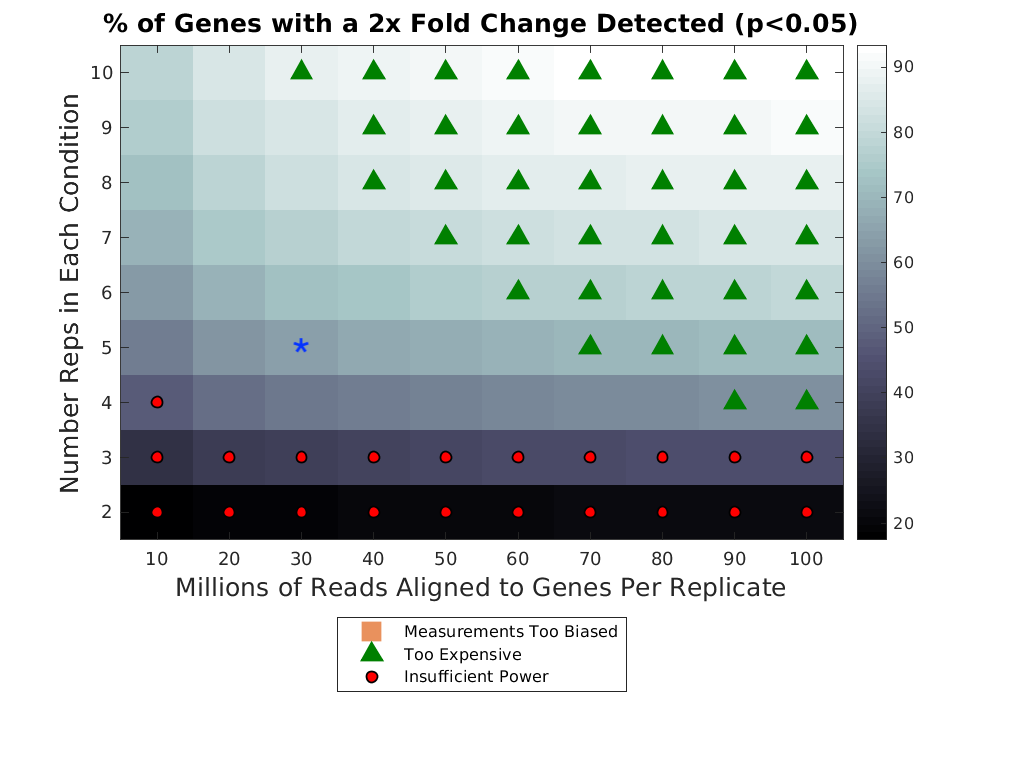
**

Fig. S4. Power analysis of differential gene expression showing the percent of genes with a 2-fold change in expression detected at p<0.05 for various study designs. The current study is indicated by an asterisk.

**Table S1.** The number of lesions, calculated from photographs at the time of sampling, were considerably lower in *M. myotis* compared to *M. lucifugus*.

| ***M. lucifugus*** | **Number of lesions** | | ***M. myotis*** | **Number of lesions** | |  |  |
| --- | --- | --- | --- | --- | --- | --- | --- |
| **ID** | **Right** | **Left** | **ID** | **Right** | **Left** |  |  |
| **8** | 3469 | 3018 | 2 | 8 | 11 |  |  |
| **11** | 1892 | 1642 | 9 | 247 | 89 |  |  |
| **16** | 1379 | 1529 | 13 | 21 | 18 |  |  |
| **21** | 1912 | 2947 | 17 | 127 | 48 |  |  |
| **23** | 1789 | 2137 | 18 | 73 | 192 |  |  |
|  |  |  |  |  |  |  |  |

Table S2. Mapping rates *to M. lucifugus* reference genome for the samples from *M. lucifugus* and *M. myotis*.

| **Sample** | **Reads** | **STAR uniquely mapped** | **Rate** |
| --- | --- | --- | --- |
| Mylu08_UVneg | 28,227,304 | 24,258,895 | 85.94% |
| Mylu08_UVpos | 31,885,284 | 27,383,551 | 85.88% |
| Mylu11_UVneg | 36,076,494 | 30,863,508 | 85.55% |
| Mylu11_UVpos | 25,449,264 | 21,912,022 | 86.10% |
| Mylu16_UVneg | 29,569,913 | 25,511,454 | 86.28% |
| Mylu16_UVpos | 28,215,949 | 24,319,333 | 86.19% |
| Mylu21_UVneg | 22,909,267 | 19,639,759 | 85.73% |
| Mylu21_UVpos | 31,351,109 | 26,883,142 | 85.75% |
| Mylu23_UVneg | 32,993,452 | 28,096,045 | 85.16% |
| Mylu23_UVpos | 30,890,733 | 26,043,495 | 84.31% |
| Mymy02_UVneg | 34,290,736 | 26,448,603 | 77.13% |
| Mymy02_UVpos | 28,020,461 | 21,775,023 | 77.71% |
| Mymy09_UVneg | 28,040,967 | 21,667,504 | 77.27% |
| Mymy09_UVpos | 30,109,388 | 23,463,240 | 77.93% |
| Mymy13_UVneg | 32,855,251 | 25,328,644 | 77.09% |
| Mymy13_UVpos | 25,009,540 | 19,414,725 | 77.63% |
| Mymy17_UVneg | 29,149,471 | 22,377,246 | 76.77% |
| Mymy17_UVpos | 27,854,272 | 21,233,176 | 76.23% |
| Mymy18_UVneg | 31,672,563 | 24,068,775 | 75.99% |
| Mymy18_UVpos | 26,030,730 | 19,637,701 | 75.44% |

Table S3. All of the *M. myotis* samples were obtained from the plagiopatagium (P), while three *M. lucifugus* biopsies were from the chiropatagium (C), all in the UV negative group.

| **Label** | **Individual** | **Group** | **Species** | **Location** | **Infection** |
| --- | --- | --- | --- | --- | --- |
| Mylu08UV- | Mylu08 | MyluUVneg | Mylu | P | UV-negative |
| Mylu08UV+ | Mylu08 | MyluUVpos | Mylu | P | UV-positive |
| Mylu11UV- | Mylu11 | MyluUVneg | Mylu | C | UV-negative |
| Mylu11UV+ | Mylu11 | MyluUVpos | Mylu | P | IUV-positive |
| Mylu16UV- | Mylu16 | MyluUVneg | Mylu | C | UV-negative |
| Mylu16UV+ | Mylu16 | MyluUVpos | Mylu | P | UV-positive |
| Mylu21UV- | Mylu21 | MyluUVneg | Mylu | P | UV-negative |
| Mylu21UV+ | Mylu21 | MyluUVpos | Mylu | P | UV-positive |
| Mylu23UV- | Mylu23 | MyluUVneg | Mylu | C | UV-negative |
| Mylu23UV+ | Mylu23 | MyluUVpos | Mylu | P | UV-positive |
| Mymy02UV- | Mymy02 | MymyUVneg | Mymy | P | UV-negative |
| Mymy02UV+ | Mymy02 | MymyUVpos | Mymy | P | UV-positive |
| Mymy09UV- | Mymy09 | MymyUVneg | Mymy | P | UV-negative |
| Mymy09UV+ | Mymy09 | MymyUVpos | Mymy | P | UV-positive |
| Mymy13UV- | Mymy13 | MymyUVneg | Mymy | P | UV-negative |
| Mymy13UV+ | Mymy13 | MymyUVpos | Mymy | P | UV-positive |
| Mymy17UV- | Mymy17 | MymyUVneg | Mymy | P | UV-negative |
| Mymy17UV+ | Mymy17 | MymyUVpos | Mymy | P | UV-positive |
| Mymy18UV- | Mymy18 | MymyUVneg | Mymy | P | UV-negative |
| Mymy18UV+ | Mymy18 | MymyUVpos | Mymy | P | UV-positive |

Supplemental Dataset 1(separate file)

Sorted and normalized differential expression of transcripts in *M. lucifugus* samples. See the second tab of the spreadsheet for the legend.

Dataset 2 (separate file)

All of the genes differentially expressed in these categories due to local infection in *M. lucifugus* showed lower fold-changes due to infection in *M. myotis* and were not differentially expressed in UV-positive tissue (Figure 3 and Dataset S3) or with respect to biopsy location (Supplemental Table 3). See the second tab of the spreadsheet for the legend.

Supplemental Dataset 3 (separate file)

Results for REVIGO analysis used to filter the gene ontology categories for redundancy.

Supplemental Dataset 4 (separate file)

When analyzing the *M. lucifugus* samples for differential expression based on biopsy location, we found strong enrichment of muscle development and function gene ontology categories. See the second tab of the spreadsheet for the legend.
